# Diatoms for Carbon Sequestration and Bio-Based Manufacturing

**DOI:** 10.3390/biology9080217

**Published:** 2020-08-10

**Authors:** Deepak Sethi, Thomas O. Butler, Faqih Shuhaili, Seetharaman Vaidyanathan

**Affiliations:** 1 Department of Chemical and Biological Engineering, The University of Sheffield, Sheffield S1 3JD, UK; faahmadshuhaili1@sheffield.ac.uk (F.S.); S.vaidyanathan@sheffield.ac.uk (S.V.); 2 School of Bioprocess Engineering, Universiti Malaysia Perlis (UniMAP), Arau 02600, Perlis, Malaysia

**Keywords:** carbon supply, CO_2_ uptake, carbon fixation, CCM, biomanufacturing, diatoms

## Abstract

Carbon dioxide (CO_2_) is a major greenhouse gas responsible for climate change. Diatoms, a natural sink of atmospheric CO_2_, can be cultivated industrially in autotrophic and mixotrophic modes for the purpose of CO_2_ sequestration. In addition, the metabolic diversity exhibited by this group of photosynthetic organisms provides avenues to redirect the captured carbon into products of value. These include lipids, omega-3 fatty acids, pigments, antioxidants, exopolysaccharides, sulphated polysaccharides, and other valuable metabolites that can be produced in environmentally sustainable bio-manufacturing processes. To realize the potential of diatoms, expansion of our knowledge of carbon supply, CO_2_ uptake and fixation by these organisms, in conjunction with ways to enhance metabolic routing of the fixed carbon to products of value is required. In this review, current knowledge is explored, with an evaluation of the potential of diatoms for carbon capture and bio-based manufacturing.

## 1. Introduction—The Carbon Calamity

Global anthropogenic activities are resulting in annual carbon dioxide (CO_2_) emissions in excess of 40 GtCO_2_ y^−1^ [1]. Over the past decade, there have been modest declines in CO_2_ emission in the USA and the 28 (now 27) European Union countries, but increasing emissions in China, India and most developing countries have dominated global emission trends, resulting in a global increase in CO_2_ emissions of 0.9% per year [2]. Even during the economic crisis of the COVID-19 pandemic in 2020, the unprecedented cessation of human activities has all but led to a small dent in the global energy use and resulting CO_2_ emissions [3]. A slowdown in CO_2_ emissions will only occur when fossil fuels, especially coal, are replaced by renewables, such as solar, wind, biomass and other sustainable alternatives, and conventional vehicles are replaced by an electric fleet that relies on renewable energy generation at point sources [2,4]. The world’s oceans are the most heavily utilized carbon storage sites, and already contain 39 trillion metric tons of carbon, where sinking particles transport carbon to the seafloor and it is buried in the sediment. There is a limit to the CO_2_ sequestration capacity of oceans, and it is projected that the pH of the oceans will further decrease by 0.3 to 0.4 units by the end of the century, which could dramatically alter marine food chains [5]. Therefore, there is an urgent necessity to develop feasible strategies for CO_2_ sequestration to alleviate the concerns.

Current strategies to reduce CO_2_ emissions include absorption, adsorption, membrane separation and cryogenic fractionation, and their limitations have been critically evaluated [6]. It has been identified that out of all the capture processes, post-combustion capture is the most relevant process that can be retrofitted to existing industrial infrastructures. The technology most explored to date for the sequestration of CO_2_ is chemical-based sequestration, but it has its own set of challenges. Recent research on carbon capture has mostly focused on optimizing CO_2_ absorption using amines, predominantly mono-ethanolamine (MEA) (a molecule developed in the 1970s), to minimize the energy consumption and to improve absorption efficiency. However, the process still remains energy intensive, and possible degradation reactions could lead to the formation of toxic compounds such as nitrosamines [7]. The ammonia-based CO_2_ capture technology can be suitably utilized only where there is residual heat for generating low grade steam used to provide the regeneration energy. Furthermore, there are common issues such as ammonia slippage [8].

CO_2_ sequestration by photosynthetic organisms can be a sustainable alternative when coupled to bioprocessing and biomanufacturing for value-addition. The photosynthetic production of molecular oxygen, otherwise known as oxygenic photosynthesis, was first observed in the ancestors of the present-day cyanobacteria, more than 2.7–3.7 billion years ago [9]. Microalgae are some of nature’s finest examples of solar energy conversion systems. They convert carbon dioxide into complex organic molecules through photosynthesis, with theoretical efficiencies in the order of 8–10% of solar energy (biomass productivities of 280 ton dcw ha^−1^ y^−1^), translating to 3% conversion efficiency in practice (biomass productivities of up to 146 ton dcw ha^−1^ y^−1^ in small scale cultivations and 60–75 ton dcw ha^−1^ y^−1^ in mass cultivations) [10,11]. It is well known that microalgae do not need arable land, and can be cultivated on marginal land, in deserts, in brackish water, or even in the open ocean, and thus do not compete with food crops for resources. Microalgae cultivations can use CO_2_ from flue gases of power stations containing SOx and NOx, and can be coupled with wastewater treatment plants for the remediation of nitrates and phosphates, heavy metals in tertiary wastewater, and for removing secondary pollutants, e.g., pharmaceuticals [12]. Microalgae have been found to have a higher CO_2_ uptake rate than forests [13]. Although large-scale microalgal cultivation for biofuels has been limited due to concerns of economic viability and sustainability, many companies are successfully producing biomass and added-value chemicals, such as pigments (β-carotene, astaxanthin, phycocyanin) and omega-3 fatty acids (docasahexaneoic acid and eicosapentaenoic acid). In addition, several companies are utilizing renewable energy for running the production plants, e.g., solar energy (AlgaTechnologies-Israel, Brevel-Israel, Simris-Sweden) and geothermal (Algalif-Iceland). The carbon content of microalgal cells typically ranges between 40–60% dcw. For a carbon content of 50% dcw, the amount of carbon potentially fixed with current biomass productivities in the range of 60–140 ton dcw ha^−1^ y^−1^ (see above) would be 30–70 ton C ha^−1^ y^−1^. This translates to a potential CO_2_ fixing capacity in the region of 100–250 ton CO_2_ ha^−1^ y^−1^. Although this would mean several hectares of cultivations to make an effective contribution to global CO_2_ mitigation, every bit of contribution adds to the total and justifies development of strategies that maximize the potential of microalgal CO_2_ sequestration.

Diatoms are a group of microalgae found in all aquatic environments, reportedly responsible for 20% of the global net primary production and 40% of marine primary production, in nature [14]. They have evolved from their ancestors, from about 250 to 190 MYA (million years ago) [15,16], and have become a highly diverse and biogeochemically relevant group of phytoplankton, and contribute significantly to the natural carbon sink [17]. Diatoms have many adaptations enabling them to thrive in the oceans. The diatomic silica cell wall may discourage ingestion by grazing organisms, provide necessary support for the large vacuole, facilitate light harvesting, increase nutrient uptake, and protect the cell against UV radiation [18]. Diatoms are favored over other phytoplankton groups in environments with fluctuating light, as occurs in non-stratified water columns, due to their favorable photo-physiology, as demonstrated for *Phaeodactylum tricornutum* [19] and *Thalassiosira weissflogii* [20]. Diatoms are well adapted to turbulence, and can be more productive in these environments compared to other microalgae [21,22]. They are an algal taxonomic group that offer a potential bio-based solution to rising CO_2_ levels. *P. tricornutum* and *Thalassiosira pseudonana* are two of the most well characterized species of diatoms. Furthermore, diatoms are very adaptive and can serve as ideal candidates for manufacturing bulk commodity products (biomass, biofuels, protein and bioplastics) and specialty chemicals (eicosapentaenoic acid, docasahexaenoic acid, fucoxanthin and recombinant proteins, e.g., recombinant antibodies) as a viable cell factory, whilst enabling strategies to reduce CO_2_ in the atmosphere [23].

This review showcases the potential of diatoms for CO_2_ sequestration, coupled with bio-based manufacturing, highlighting the challenges to be overcome for a commercially viable, sustainable manufacturing solution. A strong emphasis is given to the mechanisms responsible for carbon acquisition, transport, and processing in diatoms, as a target for improvement of carbon fixation. 

## 2. Diatoms for Bio-Based Manufacturing

Diatoms are unicellular microalgae possessing a silicon-based cell wall, and belong to the class Bacillariophyceae. They are an ecologically successful taxonomic group of phytoplankton. They contribute heavily to the global primary productivity [17,24], and play fundamental roles in the global nutrient cycling of carbon, nitrogen, phosphorus, and silicon [25,26]. The silica exoskeleton provides diatoms with structural integrity and protection in the ocean environment. Silicification increases cell density, enabling the cells to sink; possibly a selective evolutionary trait to move the cell to more optimal growth environment deeper in the water column, and evolved as a selection pressure against parasitism [27] that can be useful in establishing cost-effective harvesting methods. Their ability to prosper in the natural environment indicates their suitability for large scale cultivations in less sterile environments, to enable viable industrial scale operations. Diatoms have considerable metabolic diversity attributable to their evolution that involved endosymbiosis of diverse lineages. As a result, they can be employed to produce diverse chemicals. Manipulation of CO_2_ supply can also be used to improve the accumulation of both lipids and carbohydrates, as has been studied in *T. pseudonana*, *P. tricornutum*, *Asterionella formosa* and *Navicula pelliculosa* [28]. The presence of efficient uptake systems for CO_2_ and bicarbonate (HCO_3_^−^) have been identified in the diatoms *T. weissflogii* and *P. tricornutum*, at concentrations typically encountered in ocean surface waters. The ability to adjust uptake rates to a wide range of inorganic carbon supply has also been reported [29]. Nevertheless, there is paucity of information and evidence regarding CO_2_ uptake and there are many unanswered questions. In addition to photo-autotrophy, mixotrophic cultivation regimes can help yield higher biomass concentrations and productivities.

Diatoms can be cultivated in both indoor and outdoor settings, as suspension cultures (in open ponds, flat panel, tubular and airlift photobioreactors (PBRs)), as well as immobilized cultivation systems to avoid dewatering costs. *P. tricornutum* biomass productivity was found to be doubled in high-technology photobioreactors to 21 ± 2.3 g m^−2^ d^−1^, compared to cultivation in open ponds, and resulted in a CO_2_ fixation rate of 35.5 g^−1^ m^−2^ [30]. Overall, this gives flexibility in cultivation, as different cultivation methods can be used to enhance productivity. Novel culturing media, such as FDMed medium, have been used for high biomass, fucoxanthin and EPA production yields in freshwater diatoms, such as *Sellaphora minima* and *Nitzschia palea* in autotrophic batch cultures [31]. Media for cultivation of freshwater diatoms include: FDMed medium [31], WC [32] and modified COMBO (MCOMBO) (modified COMBO (MCOMBO)) medium of the UTEX Culture Collection of Algae). F/2 media [33], DAM (diatom artificial medium; [34]), ASW (artificial sea water; [35]), Walne media [36] can be used for the culturing of marine diatoms. Yeast extract supplementation of F/2 media has been reported to result in increased biomass concentration (3.48 fold), TAG content (2.13 fold) and fucoxanthin content (1.7 fold) in the stationary phase [37].

Optimization of operational conditions has been shown to be useful in increasing product yields. Several such studies have been reported with the model diatom, *P. tricornutum*, for example, light shift with tryptone addition to improve fucoxanthin production [38], UV mutagenesis to improve EPA productivity by 33% [39], adaptive laboratory evolution to improve neutral lipid and carotenoid accumulation [40]. Marginal improvement in total lipid contents, in association with reduced poly unsaturated fatty acids, have been observed in *Cyclotella cryptica* as a result of silicate deprivation [41]. In the case of *P. tricornutum*, a weakly silicified diatom, the required quantities of silicon can be obtained from silicon dissolved from glass vessels in alkaline culture media [42]. *P. tricornutum* grown in the presence and absence of silicon showed little difference in growth, except under low light and green light conditions [43].

In addition to their fundamental role in global nutrient cycles, diatoms represent a potential bioprocess platform, for synthesizing biofuels and other value-added products. Microalgae, in general, are of considerable interest, because many accumulate significant amounts of energy-rich compounds, such as triacylglycerol (TAG), or other lipids that can be used as biofuel precursors [44]. Diatoms have been showcased to produce both homologous and heterologous compounds, proteins and other products (Table 1). 

## 3. Carbon Assimilation in Diatoms

Carbon can be found in many forms in the natural environment. In the oceans, the dynamics of chemical dissolution of CO_2_ and its biological uptake creates an interplay between chemical and biological equilibria that requires further elucidations and understanding. For terrestrial photosynthetic organisms, atmospheric CO_2_ is the main form of inorganic CO_2_ assimilated, but in water, the dissolution of CO_2_ results in carbonic acid, which dissociates into bicarbonate and carbonate. In the oceans, 90% of inorganic carbon is in the form of bicarbonate [80]. Prior to the industrial revolution, CO_2_ concentrations in the atmosphere were ~280 ppm [81], but today they have increased to ~420 ppm in 2020 (https://www.co2.earth/), with an increasing proportion of CO_2_ sequestered in the oceans and on land. At pre-industrial concentrations of atmospheric CO_2_, the seawater concentration of bicarbonate was 1757 µmol kg^−1^, but elevated levels of bicarbonate are now being observed, contributing to ocean acidification and a higher solubility of carbonate [80]. 

The effect of increasing CO_2_ concentration supply to diatoms leads to increased growth and biomass production, under growth optimal conditions. Carbon capture in *P. tricornutum* happens predominantly in the form of bicarbonates with bicarbonate transporters [82], and as mentioned above, CO_2_ fixation rates of 35 g m^−2^ d^−1^ have been reported [30]. When cultivating *P. tricornutum* in air sparged cultures, a CO_2_ consumption rate of 1 g g^−1^ DW, at pH 7.2 and 0.8 g g^−1^ DW, at pH 9, both resulting in 0.06–0.08 g CO_2_ uptake per day (removal of 50–65% of CO_2_ from the air), has been reported [83]. It has been identified that the optimal CO_2_ concentration for biomass accumulation is in a narrow range, between 1% and 1.25% CO_2_ in air (v/v), at a gas supply rate of 0.66 vvm and light intensity of 1000 µmol m^−2^ s^−1^ (16 h light period), 90% of CO_2_ supplied leaving the medium unused [84]. When *P. tricornutum* was provided with bicarbonate as an inorganic carbon source, between 73–99.9% of the bicarbonate was consumed or remained dissolved in the medium, resulting in a CO_2_ consumption rate of 0.31 g d^−1^ (2.3 g CO_2_ g^−1^ biomass), albeit at the cost of reduced growth and biomass production [83]. Cultivations of *P. tricornutum* (PHAEO2) in modified F/2 seawater (enriched four-fold with nitrogen and phosphorus) with 15% CO_2_ have been shown to increase biomass productivity to 0.15 g L^−1^ d^−1^, whilst consuming 0.28 g L^−1^ d^−1^ of CO_2_ in a batch operation [85]. A comparative assessment of CO_2_ concentration mechanisms (CCMs) in a handful of freshwater and marine diatoms (*P. tricornutum*, *As. formosa*, *N. pellicosa*, *T. pseudonana*, *T. weissflogii*) revealed that, for all the species, at 20,000 ppm, the affinity for DIC was lower than at 400 ppm CO_2_ (atmospheric concentrations), and the reliance on CO_2_ was higher, and that species-specific differences were greater than environmental differences, in determining the effectiveness of the CCMs [86]. Negative effects of CO_2_ on growth have also been recorded. For example, *Attheya longicornis* growth was hampered by high levels of CO_2_ supply [87]. Another factor affecting marine species is temperature. Rising temperatures may also have a negative effect on the CO_2_ uptake rate by diatoms. In *Navicula distans*, rising temperature and pCO_2_ resulted in a reduction of diatom cell size, which inevitably relates to the ecological and physiological functions of diatoms, such as nutrient diffusion, intake and requirements, and even the metabolic rate [88]. There are also some cases recorded where no reaction to increased CO_2_ levels could be observed, as seen with *Chaetoceros brevis* cultures supplemented with pCO_2_ (750 ppmv (2 × ambient) and 190 ppmv (0.5 × ambient) CO_2_), where little or no significant effect was observed on the diatom growth, pigment content and composition, photosynthesis, photoprotection and RuBisCO activity [89]. 

**Figure 1:**
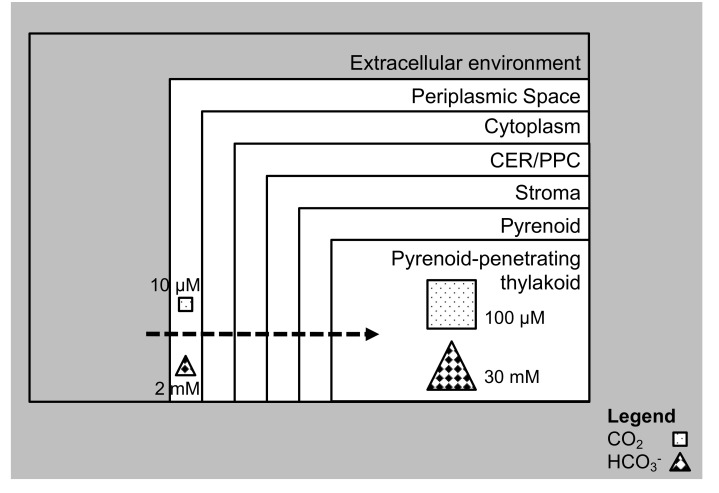
Carbon dioxide (CO_2_) enrichment in cell organelles. In diatoms, there are different membranes for CO_2_ to cross, and it has to be enriched from low to concentrated levels near RuBisCO to enable CO_2_ fixation. The periplasmic space faces the extracellular environment. Adjacent to the periplasmic space is the cytoplasm. Further inward is the chloroplastic endoplasmic reticulum (CER)/periplastidal compartment (PPC). The stroma is the layer beyond the CER/PPC. The innermost layer is the pyrenoid where in embedded is the pyrenoid penetrating thylakoid. This usually happens in general, with CO_2_ concentration mechanisms (CCM) in microalgae.

CO_2_ uptake in the aquatic photosynthetic organisms, such as diatoms, cyanobacteria and other microalgae, take place with the involvement of the CCM. Carbon metabolism pathways in diatoms, like in plants and other algae, require the transportation of CO_2_ across intracellular compartments like the peroxisomes, chloroplasts, mitochondria, endoplasmic reticulum and the cytosol, with concentration at the site where RuBisCO is located for CO_2_ fixation (Figure 1). This arrangement gives flexibility to the cell to adjust the carbon flux, enhance the concentration of CO_2_ in a stepwise manner from low concentrations on the outside to levels required for RuBisCO activity and hence fix CO_2_ [90,91]. There is limited information available on carbon metabolism in several diatoms, as of now. In order to obtain the appropriate design of the carbon flow in diatom cells under different conditions, information regarding the localization and functionality of the component diatom enzymes is a necessity. The cellular machinery involved in diatom photosynthesis includes the chloroplasts, carbonic anhydrases (CAs), RuBisCO, Calvin Benson Bassham (CBB) cycle proteins, transporters, phosphoglycerate kinase (PGK), glyceraldehyde-3-phosphate dehydrogenase (GAPDH), CP12, fructose-1,6-biphosphatase (FBPase), sedoheptulose-1,7-biphosphatase (SBPase), phosphoribulokinase (PRK), basic leucine zipper (bZip) bZIP transcription factors family, and others [24,82,86,92,93]. Diurnal rhythms also affect the TCA Cycle and that influences the amount of CO_2_ that is absorbed. Moreover, the bZIP14 protein family members are involved in CO_2_ sensing and blue light signaling [94]. Our current knowledge of diatom CCMs is discussed in the section below, an understanding of which will help in devising strategies to maximize uptake of CO_2_ by diatoms.

### 3.1. The Diatom CCM and the Chloroplast Pump Model

The physical constraints on photosynthesis in the marine environment, especially low (dissolved) CO_2_ in seawater, is partially mitigated by the CCM. Photosynthetic CO_2_ fixation was found to be near saturation at external concentrations of 200 µM (as bicarbonate and CO_2_) [95]. The CCM of *P. tricornutum* is reported to be moderately efficient, with around one-third of the carbon transported into the chloroplast being fixed by RuBisCO, and the remainder leaking out as CO_2_, attributable to the limited permeability of diatom membranes to CO_2_. The major driver of the CCM is believed to be the chloroplast pump, which actively transports bicarbonate into the chloroplast, where the bicarbonate flux into the chloroplast exceeds the net CO_2_ and bicarbonate flux across the plasmalemma. Additional bicarbonate transporters are required in the membranes surrounding the chloroplast [82]. A large inorganic carbon pool is accumulated in the chloroplast, hence elevating CO_2_ concentrations around RuBisCO and inorganic carbon is depleted from around the cytoplasm resulting in a diffusive influx of CO_2_ from the extracellular environment into the cytoplasm [96]. Some of the proteins responsible for bicarbonate transport are embedded in the chloroplast membrane, but the full characterization of these proteins is ongoing. It is hypothesized that a different solute career 4 (SLC4) family of transporters in sequence move bicarbonate from the external environment to the chloroplast stroma [82].

**Figure 2:**
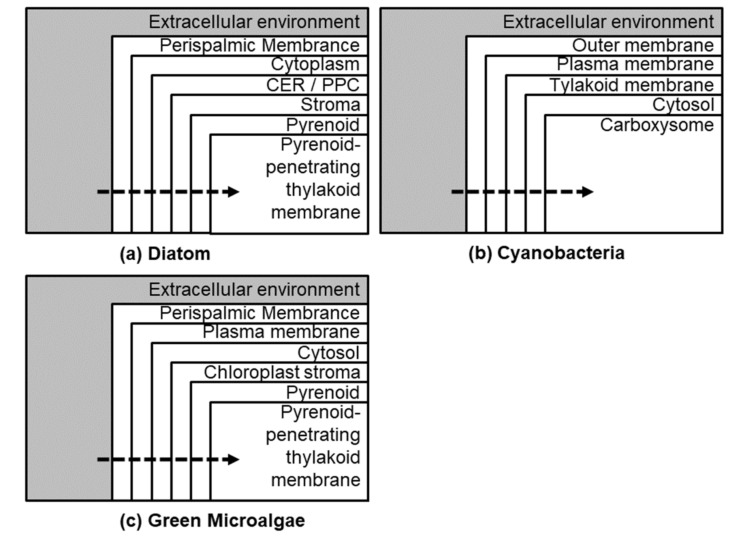
Broad differences (representative) between diatom CCMs (**a**) with that of cyanobacteria (**b**) and green algae (**c**).

**Figure 3:**
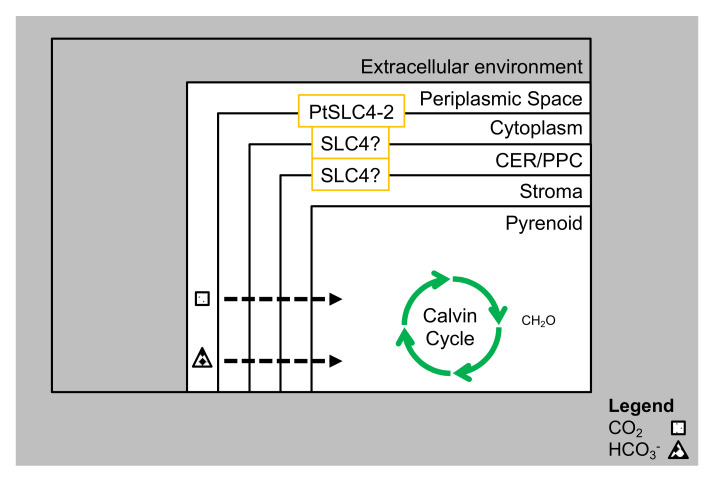
CCM with transporters in *P. tricornutum.* The thin arrows denote diffusion of CO_2_ through the membranes, while the broad arrows denote active transportation of HCO_3_^−^ by SLC4s (Solute Carrier Family 4) transporters.

**Figure 4:**
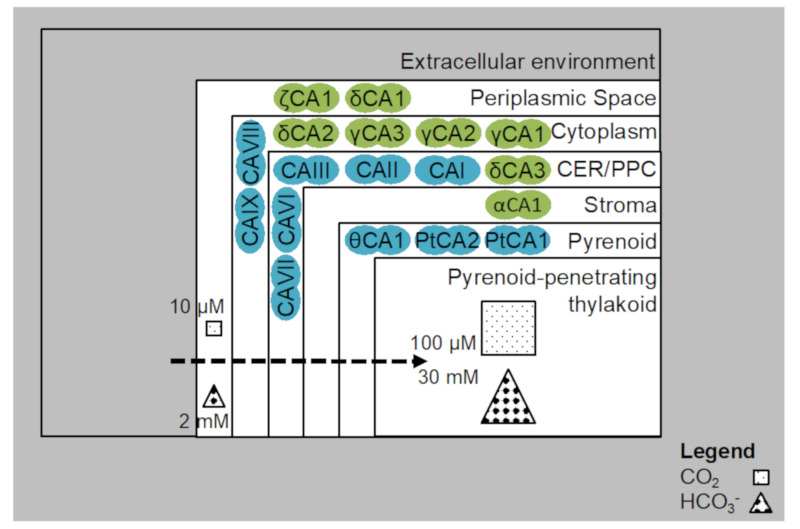
Distribution of CAs in model species: *P. tricornutum* (blue), *T. pseudonana* (green). CO_2_ is enriched from 10 μM in the outermost layers to 100 µM in the innermost layers of the pyrenoid. HCO_3_^−^ is enriched from 2 mM in the outermost layers to 30 mM in the innermost layers of the pyrenoid. There is interconversion involved between CO_2_ and HCO_3_^−^ in the outermost and innermost layers also [82].

Cyanobacteria and green algae, such as *Chlamydomonas reinhardtii*, developed CCMs (Figure 2), to reduce the impact of the oxygenase activity of RuBisCO. The most characterized eukaryotic CCM is that found in *C. reinhardtii*. Inorganic carbon (as bicarbonate) is pumped into the chloroplast by active transport, where it is converted to CO_2_ by carbonic anhydrase (CA), CAH3 (localized in the thylkaoid lumen). The stromal soluble protein complex (CrLCIB-LCIC) has the ability to re-capture and to prevent the leakage of CO_2_ generated by CAH3. Moreover, CrLCIB-LCIC is not fixed by RuBisCO [97]. *C. reinhardtii* actively transports both CO_2_ and HCO_3_^−^ across the plasmalemma, but CO_2_ appears to be the preferred form. Proton leakage through the thinner diffusive boundary layer is important in smaller organisms that have a smaller size [98]. The basic features of a cyanobacterial CCM include transport of inorganic carbon and the presence of carboxysomes that help in minimizing CO_2_ leakage (Figure 2). The induction of the CCM takes place at low CO_2_ levels. DIC transporters are involved in maintaining the supply of CO_2_ to RuBisCO and CAs are utilized for DIC accumulation. Diatoms use a CCM to overcome the difficulties of CO_2_ limitation in alkaline and high-salinity seawater, by using SLC4 family transporters to take up HCO_3_^−^ actively from the surrounding seawater, leading to the intracellular accumulation of DIC [99,100]. Multiple CAs maintain pH within each of the organelles, by maintaining a fine ratio of CO_2_ and bicarbonate (Figure 2, Figure 3 and Figure 4).

### 3.2. Carbon Transport Systems

Diatoms can actively take up HCO_3_^−^ and/or CO_2_ and the uptake of DIC across the plasma membrane is the critical first step in using DIC for photosynthesis [101]. The most well studied CCMs in diatoms are in *P. tricornutum* and *T. pseudonana* (Figure 4). Adequate flux of CO_2_ to ensure an optimal CO_2_ to RuBisCO ratio (amount of CO_2_ as substrate for the RuBisCO molecule) is generated and is facilitated by two pyrenoidal β-carbonic anhydrases (PtCA1 and PtCA2) [96,102,103]. The amount of CO_2_ is complemented by α-type CAs that are located in the vicinity and in the sub-cellular spaces of the four-layered chloroplast membranes, which prevent the leakage of CO_2_ from the chloroplast in *T. pseudonana* and *P. tricornutum* [103,104].

In a diffusion-based CO_2_ uptake system, HCO_3_^−^ uptake is by plasma membrane based SLC4, and CO_2_ is taken up from the external environment directly through the cell membranes, as they are permeable, and CO_2_ cannot passively pass through a transporter. CO_2_ can only be taken up by generating a CO_2_ deficit inside the cell through a diffusive mechanism, which leads to the suction of CO_2_ from the external environment [86]. The active transport of HCO_3_^−^ out of the cytoplasm and into the chloroplast leads to the formation of a low HCO_3_^−^ concentrated environment in the cytoplasm of diatoms. The action of a cytoplasmic CA leads to a reduction in the cytoplasmic CO_2_. The conversion of CO_2_ to HCO_3_^−^ occurs when the HCO_3_^−^ concentration is below equilibrium with CO_2_. The CO_2_ gradient leads to its passive diffusion into the cell across the plasma membrane, and the continued export of HCO_3_^−^ from the cytoplasm maintains a constant cytoplasmic CO_2_ deficit, resulting in sustained CO_2_ uptake. In order to maintain the inward CO_2_ suction, the activities of the transporters exporting HCO_3_^−^ from the cytoplasm should be more than that of CO_2_ and HCO_3_^−^ influx into the cell [101].

The SLC4 family have been found to represent a major group of bicarbonate transporters which have an important role in pH regulation. It has been found that the N-terminal (Nt) domain (involved in functional regulation of transporters) accounts for 32–55% of the entire polypeptides of the SLC4 transporters. Interestingly, the SLC4 like transporters in plants lack the large Nt domain found in mammalian homologs, which has been found to be non-essential for the transport of anion exchangers of the SLC4 family [105]. Bicarbonate transport at the molecular level has predominantly been studied in the model diatoms *P. tricornutum* and *T. pseudonana* [82,106]. Ten putative bicarbonate transporters have been identified in *P. tricornutum*, which are similar to those found in mammalian protein families (SLC4 and SLC26) [80]. 

Three different transport systems can be identified in diatoms (plasma membrane, plastid and aquaporin). The first is the plasma membrane-based bicarbonate transport system (PtSLC4). Under CO_2_-limiting conditions, PtSLC4-2 in the plasmalemma is induced, and its expression increases with DIC uptake and photosynthetic activity. In the presence of a high concentration of sodium ions, PtSLC4-2 transports bicarbonate and it has a saturation limit of ~100 mM sodium ions. PtSLC4-1 appears to be a sodium dependent bicarbonate transporter, and its function was inhibited by the addition of an anion-exchanger inhibitor. The bicarbonate uptake rate of PtSLC4-2 is highest at pH 8.2, equivalent to the pH of seawater [100]. PtSLC4-2 is involved in the direct uptake of bicarbonate, and is actively involved in acquisition of extracellular DIC under low CO_2_ conditions, in *P. tricornutum*. SLC4 homologs have also been characterized in *T. pseudonana* [100]. Two putative bicarbonate transporters, PtSLC4-1 and PtSLC4-4, are found to be highly conserved in comparison to PtSLC4-2. PtSLC4-1 and PtSLC4-4 are also induced specifically under CO_2_-limiting conditions, and it has been suggested that these transporters contribute heavily to bicarbonate influx into the cell in seawater in a CO_2_-limiting environment [101,107]. Other SLC4 homologs are currently being studied in the chloroplast membranes of *P. tricornutum*, and more types of transporters are awaiting identification of location and functionality [101]. It has been suggested that different SLC4 transporters translocate bicarbonate from the environment to the chloroplast stroma [82]. A different group of SLC4 transporters in the chloroplast envelope are thought to transport bicarbonate to the chloroplast stroma [80]. 

In plastid membrane-based HCO_3_^−^ transport systems, the DIC that is imported into the cytosol is not able to freely diffuse into the chloroplast for fixation. This is due to the chloroplast membrane, which is four-layered. At each of the four-layers, HCO_3_^−^ transporters should be present to regulate the transport, aided by the CAs that should be present in close proximity to the chloroplast membranes and the transporters [96,101]. However, such HCO_3_^−^ transporters are only proposed, and yet to be identified in diatoms. From genome annotations in *P. tricornutum*, it has been elucidated that there are three putative PtSLC26 genes, and seven PtSLC4 genes [100]. This displays the limited information regarding its location, structure, and the overall functionality in the scheme of carbon transport within the system. PtSLC4-6 and PtSLC4-7 genes have been found to be active and upregulated under high CO_2_ and low CO_2_ conditions [100], implying that PtSLC4-6 and PtSLC4-7 act as DIC transporters, and are involved in the regulation of the DIC flow from the cytosol to the plastid, and remains unaffected by the ambient CO_2_ levels. Presently, the information regarding the mechanism of activity of the DIC transporters, such as PtSLC4-6 and PtSLC4-7, still remains highly limited [101].

CO_2_ permeation through diatom membranes is very rapid [102], and this high permeability may be, in part, due to the presence of channels such as aquaporins (AQPs) [101]. In the aquatic environments, AQPs are ubiquitous water channels that have been known to facilitate the transport of many small molecules such as CO_2_ and ammonia. AQPs are involved primarily in mechanisms that are responsible for maintaining the transmembrane fluxes of important small molecules, that are yet to be studied in marine photoautotrophic organisms. *In silico* analysis has revealed the presence of five AQP orthologs in *P. tricornutum* and two in *T. pseudonana* [108]. It is also believed that lipid bilayers being inherently permeable to CO_2_, also do present some resistance to diffusion that could be reduced by the presence of AQPs, as a result of which DIC can be easily imported into the cell and fixed, albeit with minimal energy expenditure.

### 3.3. Carbonic Anhydrase-Isoforms and Activation

The primary function of CAs is to interconvert CO_2_ and bicarbonate, to enable transport across membranes and prevent loss of CO_2_. In addition, they are responsible for recovering CO_2_ leaked from the chloroplast. This implies that a CCM is primarily regulated by CAs. CO_2_ uptake is due to the internal CO_2_ deficit generated by CA-catalyzed hydration of CO_2_ to HCO_3_^−^ in the cytoplasm [101]. CO_2_ uptake by its synthesis from HCO_3_^−^ at close proximity to the cell surface can also be done by external CAs, located in the periplasmic space [109]. There is a complex plethora of possible carbonic anhydrase isoforms from diatoms. They are found in diatoms, with varying capabilities in terms of carbon absorption, assimilation and utilization. All of the identified CAs are at different stages of confirmation, with respect to localization, functionalization and identity [82]. Ten putative CA genes have been identified in *P. tricornutum* [110], five α-CA, two β-CAs, two γ-CAs and one θ-CA [103,111]. Notably, α-CAs are located at the four-layered chloroplast membrane, β-CAs in the pyrenoid, γ-CAs in the mitochondria and the θ-CA in the thylakoid lumen. CA activity has only been verified with two β-CAs and one θ-CA [101]. There are no free stromal CAs, no external and no cytosolic CAs identified in *P*. *tricornutum* [111].

CAs are metallo-enzymes, and zinc is essential for their activity [80]. However, several coastal diatoms have cadmium containing CAs, and this is considered an evolutionary adaptation to low zinc in marine habitats, but the cadmium at the catalytic site of the ζ-CA can be exchanged for zinc [112,113]. CA enzymes are ubiquitous in nature, and provide an example of convergent evolution. They appear to have a diverse role in many biological processes, including CO_2_ fixation, pH homeostasis, and the transport of CO_2_/bicarbonate. Seven distinct classes of CAs have been identified, i.e., α, β, γ, δ, ζ, η, and θ-Cas, out of which α, β, γ CAs are found in higher land plants, but δ and ζ CAs are restricted to marine diatoms [80]. The θ-CA has been found to be widely distributed in algae and cyanobacteria, and it has been reported to be essential for photosynthesis in *P. tricornutum* [111].

Two pyrenoidal β-carbonic anhydrases (CAs) have already been isolated and characterized in *P. tricornutum* [102,103,114], but the actual reason for the existence of different CAs is not known, but the diversity could be due to different locational requirements. New subsets of CAs in *T. weissflogii* and *T. pseudonana*, have been identified. They are δ-CAs, ζ-CAs and θ-CAs [111,113,115,116,117]. It is predicted that *P. tricornutum* lacks periplasmic CAs, however surprisingly possesses an efficient CCM, which may suggest that periplasmic CAs are not necessary for the operation of a CCM in microalgae [82]. Furthermore, diatom-specific adaptations in chloroplast metabolism highlight beneficial traits. These include the completion of tocopherol synthesis via a chloroplast-targeted tocopherol cyclase, a complete chloroplast ornithine cycle, and the chloroplast-targeted proteins involved in iron acquisition and CO_2_ concentration not shared between diatoms and their closest relatives in the Stramenopiles family [118].

Ambient CO_2_ is required to trigger the transcription of *ptca1*, and light affects the extent of acclimation [119]. An appropriate combination of CO_2_/cAMP-responsive elements i.e., CCRE1/2 or CCRE2/3 at proper distances from the minimal promoter are required as a potential target of the Zip protein PtbZIP11 for an effective CO_2_ response of the *ptca1* gene [120]. The detailed analysis of the promoter region of *ptca2* appears to indicate that both CCRE2s are cis-elements governing the CO_2_/light response of *ptca2* promoter [120]. The transcriptional activation of the two *ptca* promoters in CO_2_ limitation was evident under illumination with a photosynthetically active light wavelength. An artificial electron acceptor from the reduction side of PSI efficiently inhibited *ptca* promoter activation, while neither inhibition of the linear electron transport from PSII to PSI, nor inhibition of ATP synthesis, showed an effect on the promoter activity, strongly suggesting a specific involvement of the redox level of the stromal side of the PSI in the CO_2_/light cross talk [121].

### 3.4. Pyrenoid Matrix

The pyrenoid is a protein body containing RuBisCO found in most algal chloroplasts (in the stroma) and RuBisCO is often coated with a starch sheath. Pyrenoids are associated with operation of CCM. The main function of pyrenoids is to act as centers of CO_2_ fixation, generating and maintaining a CO_2_ rich environment around RuBisCO. Bicarbonate supply could be used to elevate CO_2_ reacting with CA in the pyrenoid [92]. In diatoms, pyrenoids are generally present, but in some species, their presence can be variable, even within a single genus. 

There is a strong correlation between the presence of a pyrenoid and an active CCM in algae [92,100]. However, the presence of a pyrenoid does not always confirm the operation of a CCM. Of all the microalgae examined in any detail, it is clear that those with the highest affinity for CO_2_ and clear CCM characteristics have a pyrenoid, and most probably a single chloroplast per cell. This is true of both the green and non-green algae. In the pyrenoid, bicarbonate is converted into CO_2_, resulting in a localized elevation in CO_2_ concentration, favoring carboxylation by RuBisCO, over oxygenation. The three-dimensional structures of the chloroplast-pyrenoid in *C. reinhardtii* have been revealed, using *in situ* cryo-electron tomography [122]. It has been found that some of the thylakoid membrane can penetrate into the pyrenoid, called the pyrenoid-tubules and may have a role in the carbon capture process. The diatom, *P. tricornutum*, has a pyrenoid-based CCM, and contains a cluster of the genes homologous to *C. reinhardtii* (LCIB), PtLCIBI-4 [97].

As carbon availability is often the limiting factor for microalgal growth, most microalgal chloroplasts contain a pyrenoid with a high concentration of RuBisCO, for an effective CCM [123]. In addition, CO_2_ responsive CAs occur in the pyrenoid of *P. tricornutum* [103]. For CO_2_ fixation by RuBisCO, the transported HCO_3_^−^ has to be converted to CO_2_. CO_2_ can also be produced in *P. tricornutum*, by importing HCO_3_^−^ into the pyrenoid-penetrating thylakoid lumen. There, the activity of θ-CA and the low pH converts HCO_3_^−^ to CO_2_ for RuBisCO. β-CAs can also convert bicarbonate into CO_2_, thereby increasing the CO_2_ concentration around RuBisCO [101]. The amount of CO_2_ supplied to RuBisCO is fixed, but the rest of the CO_2_ leaks out of the chloroplast [96]. The leaked CO_2_ is recovered by CA-catalyzed conversion to HCO_3_^−^ [82]. *P. tricornutum* lacks cytosolic and stromal CAs, but has numerous chloroplast envelope CAs. Such a design implies that the main recovery points of carbon are in the four-layered chloroplast envelope [103]. The essential pyrenoid component 1 (EPYC1) is a low complexity repeat protein which links RuBisCO to form the pyrenoid. EPYC1 is of comparable abundance to RuBisCO, and colocalizes with RuBisCO throughout the pyrenoid. EPYC1 is essential for normal pyrenoid size, number, morphology, RuBisCO content and efficient carbon fixation at low CO_2_. The most abundant proteins in the low CO_2_ pyrenoid fraction are RuBisCO large (rbcL) and small (rbcS) subunits, and RuBisCO activase (RCA1) [124]. It has been found that pyrenoid-based CCM emerges as the most effective in achieving the greatest elevation of CO_2_ [92]. CCMs increase the cellular CO_2_ concentration around RuBisCO, resulting in a higher carbon fixation rate in a CO_2_ limited environment. To avoid carboxylation/decarboxylation, and to ensure compartments in which CO_2_ can be concentrated for carbon fixation by RuBisCO, diatoms may utilize their pyrenoid and four-layered plastids [125].

### 3.5. RuBisCO and Its Activation: The Effect of Glycolate

CO_2_ is of limited supply to marine phytoplanktons, because of the low partial pressure of CO_2_ in the atmosphere, a faster CO_2_ hydration rate compared with bicarbonate dehydration rate, and the high salinity and alkalinity of seawater [125]. RuBisCO in diatoms have a CO_2_ half saturation constant of 23–68 µM [126]. The concentration of CO_2_ in seawater is 10–15 µM at pH 8.2, and therefore diatoms are thought to operate a CO_2_-CCM, to improve the efficiency of carbon fixation [125]. Photosynthesis requires the carboxylation of RuBP by RuBisCO, but photorespiration occurs when RuBisCO oxygenates, RuBP forming the toxic by-product glycolate which needs to be removed by the cell. Glycolate accumulation, as a result of photorespiration, appears to influence RuBisCO activity more than the depletion of its substrates (CO_2_ or RuBP) [127]. Photorespiration has been found to reduce the photosynthetic efficiency by 20–50% in C3 crops [128]. RuBisCO requires the concentration of CO_2_ to be more than 25 µM for carbon fixation, but the rate of conversion of HCO_3_^-^ to CO_2_ is generally found to be slow [92,126]. The catalytic step involving RuBisCO is the rate limiting step in the Calvin-Benson-Bassham (CBB) cycle. RuBisCO catalyzes the carboxylation of ribulose-1,5-bisphosphate (RuBP), to synthesize two molecules of 3-phosphoglycerate (PGA). RuBisCO has a very low affinity for CO_2_. Unlike other enzymes, the concentration of RuBisCO does not change the flux towards CO_2_ fixation. The process is affected by the relative concentration of CO_2_ and O_2_ at the active site. The rate of CO_2_ diffusion is low in aquatic systems, and the CO_2_ concentration is often below the required threshold of RuBisCO. The ratio of CO_2_ fixation rate and the photosynthetic electron transport rate regulate RuBisCO activity. For catalysis to occur, RuBisCO should be activated first. This happens with the help of RuBisCO activase (RCA). Otherwise, CO_2_ binds at a lysine residue in RuBisCO for carbamylation. A change in the conformation of RuBisCO is brought about when RCA binds to the inactive RuBisCO and ATP hydrolysis occurs. This results in the synthesis of a highly active form of RuBisCO, based on cellular requirement.

To maximize the performance of RuBisCO in photosynthetic CO_2_ fixation, the kinetic properties of the enzyme have evolved over time. RuBisCO is a highly diverse biomolecule, four forms of which have been identified to date (I, II, III and IV), categorized based on the differences in the primary polypeptide sequence, along with the differences in the number of small and large subunits. RuBisCO form I is the most common form of RuBisCO found in nature. RuBisCO form I is further sub-divided into four subforms (A, B, C and D), as a result of their evolution. Forms IA and IB (“green-type”) are found in higher plants, cyanobacteria, chlorophyceae and streptophytes [129,130], whilst forms IC and ID (“red-type”) found in haptophytes, cryptophytes, rhodophytes and the heterokontophytes [129,131]. Which form of RuBisCO is better is a matter of further research, as both the RuBisCO types have their own preferences in terms of CO_2_ needs in the environment. Currently, red algal RuBisCO is being studied in depth for crop improvement strategies, and is assumed to have better kinetics [132].

RuBisCO enzymes from microalgae have evolved a higher affinity for CO_2_ when the algae have adopted a strategy for CO_2_ fixation that does not utilize a CCM. This appears to be true for green and red algae form I RuBisCO enzymes. However, the red form I RuBisCO enzymes present in non-green algae appear to have reduced oxygenase potential at ambient concentrations of O_2_. This has resulted in a photosynthetic physiology with a reduced potential to be inhibited by O_2_ and a reduced need to deal with photorespiration. Red form I RuBisCO enzymes appear to achieve superior kinetic characteristics when compared with the RuBisCO of C3 higher plants, which are derived from green algal ancestors [92]. The green-type RuBisCO activase functions as a canonical hexameric AAA + ATPase [133,134], and its higher plant homologs mostly occupy far larger polydisperse oligomeric states. RuBisCO catalyzes the fixation of atmospheric CO_2_ in photosynthesis, but tends to form inactive complexes with its substrate ribulose 1,5-bisphosphate (RuBP). In plants, RuBisCO is reactivated by the AAA + ATPases (associated with various cellular activities) protein RuBisCO activase (Rca), but no such protein is known for the RuBisCO of red algae [135]. Understanding RuBisCO activation may facilitate efforts to improve CO_2_ uptake and biomass production by photosynthetic organisms, by making more carbon available to the system and inducing utilization, being incorporated later into biomass.

The active site of the RuBisCO molecule is formed from the N-terminus of one of the monomers and of the C-terminus of another subunit, therefore having four dimers arranged together, in an optimum conformation with ideal structural stability [130,136,137]. It is also noteworthy that the conformational changes affect the specificity factor (CO_2_/O_2_) of the RuBisCO enzyme, which is an important kinetic parameter. Premature binding of RuBP and other molecules such as other sugar phosphates inhibits RuBisCO activity; the biomolecule is also dogged by slow turnover rate, competition from oxygenase activity and low affinity towards CO_2_ [129,138]. Coccolithophores and diatoms, as a virtue of their evolutionary process, have obtained the highly selective Rhodophyte form of 1D RuBisCO. The 1D RuBisCO form is better adapted to the oxygenic environment with a higher O_2_/CO_2_ ratio. Furthermore, the 1D RuBisCO form is economical, and requires lower energetic or nutrient investment in a CCM to obtain high carboxylation rates under environmentally high O_2_/CO_2_ ratios [139]. The CCM of diatoms are highly diverse, and are capable of concentrating very high levels of CO_2_ in the pyrenoid. Diatom RuBisCO also displays very high variation in Michaelis constant for CO_2_, *K*_C_ (23–68 μM), specificity for CO_2_ over O_2_, S_C/O_ (57–116 mol mol^−1^), and Michaelis constant for O_2_, *K*_O_ (413–2032 μM), in comparison to plant and other algal RuBisCOs [126].

### 3.6. Evidence of C4 Metabolism

There are three main carbon fixation mechanisms employed by photosynthetic organisms: C3, C4 and Crassulacean acid metabolism (CAM), which have been extensively reviewed elsewhere [80,140]. The C4 type photosynthesis, as a carbon concentrating mechanism, has evolved more than 60 times, to address the inefficiencies of the ancestral C3 photosynthetic pathway. Inherent in the C4 pathway is a high rate of photosynthesis at low levels of stomatal conductance, especially at the low levels of CO_2_ in the atmosphere [141].

Less than 4% of terrestrial plant species are believed to have a C4 pathway. The C4 pathway involves a CO_2_-CCM around RuBisCO, eliminating the oxygenase function of RuBisCO, and reducing the wastage of carbon assimilation to photorespiration [125]. The operation of a C4 pathway overcomes the tendency of RuBisCO to fix oxygen rather than CO_2_, and avoids the production of glycolate, thus minimizing photorespiration. C4 plants provide a CO_2_ pump which results in an increased CO_2_/O_2_ ratio at the site of RuBisCO, resulting in decreased oxygenase activity and RuBisCO operates at close to its *V*_max_, whereas RuBisCO in C3 plants only operates at around 25% of the *V*_max_ [142]. RuBisCO in C4 plants is more efficient in terms of carboxylation than C3 plants; a higher light harvesting efficiency is observed as saturation under high light is avoided and higher nitrogen utilisation is observed, because less RuBisCO, and thus nitrogen, is required. The C4 CCM pathway in higher plants uses PEP to catalyze the first reaction in inorganic carbon fixation and requires compartmentalization called Kranz anatomy (bundle sheath and mesophyll areas). Comparatively, in single-celled plants, dimorphic chloroplasts contain a central compartment and peripheral chloroplasts. In C4-containing single-celled plants, the release of CO_2_ in the direct vicinity of RuBisCO is critical for the activity of an efficient C4 photosynthesis [125]. Understanding microalgae with a C4 mechanism could enable further developments to improve photosynthesis and allow cultivation under more extreme conditions. 

Of the few diatoms that have features of a biochemical CCM, *T. weissflogii* and *P. tricornutum* are the only ones that have been investigated in detail, to determine if they undergo C4 photosynthesis. *P. tricornutum* has a naturally slow rate of photorespiration. *T. pseudonana* and *P. tricornutum* both possess several carboxylases and decarboxylases, which could be used for a C4-like CCM. Both diatoms have a pyruvate phosphate dikinase (PPDK), which converts pyruvate into PEP, the initial three carbon molecule that accepts HCO_3_^−^ in the C4 CCM of higher plants [125]. In low CO_2_ concentrations, *T. pseudonana* has been found to use a “closed loop biochemical model”, an atypical C4-type CCM, where the back-reaction of the pyruvate carboxylation was presumed to be responsible for CO_2_ release in the plastid [93]. During the transition from growth to lipid accumulation, pyruvate carboxylase in the mitochondrion is utilized as the primary inorganic carbon fixation stage in a C4 pathway, and malate undergoes decarboxylation by the malic enzyme in the peroxisome to concentrate CO_2_ for diffusion into the chloroplast [143]. 

Whilst genome analysis has indicated that *P. tricornutum* possesses the necessary enzymes for operating a C4 pathway, recent evidence has suggested that a C4-like CCM is not present [125]. Neither radiolabeling with ^14^C nor PPDK silencing experiments via RNAi with the aim of identifying primary products of inorganic carbon fixation were found to support the presence of a C4-like CCM in *P. tricornutum*, *T. pseudonana*, *T. weissflogii*, *As. Formosa*, *N. pelliculosa* [86]. None of the known or investigated decarboxylases nor a cytosolic PEP carboxykinase (PEPCK) has been found in the plastid (a prerequisite for a single cell C4-type CCM pathway). The CCM efficiency of *P. tricornutum* is not affected by a reduction in PEPCK activity, and therefore PEPCK does not appear to contribute to the CCM. It has been concluded that the C4-like CCM in *P. tricornutum* does not have an essential role in CO_2_ fixation, and the enzymes investigated are more likely involved in similar functions (e.g., gluconeogenesis, amino acid synthesis or replenishing the TCA cycle), seen in C3 plants [125].

In photosynthesis, the efficient conversion of CO_2_ into organic matter requires a tight control of the ATP/NADPH ratio which, in other photosynthetic organisms, relies principally on a range of plastid-localized ATP generating processes. Diatoms can regulate ATP/NADPH through extensive energetic exchanges between plastids and mitochondria. This interaction comprises the re-routing of reducing power generated in the plastid towards mitochondria and the import of mitochondrial ATP into the plastid, and is mandatory for optimized carbon fixation and growth [144]. 

In summary, as can be seen from the molecular mechanisms highlighted above, our knowledge of CO_2_ fixation in diatoms is informed largely by investigations carried out in a handful of diatoms, and extrapolated from plants and other microalgal species. Given the diversity in diatoms and their metabolic capabilities, molecular level data from other diatoms under specific industrial CO_2_ sequestering environments would be required to develop a broader picture of the diatoms CO_2_ utilization capabilities. Nevertheless, the adaptive nature of these organisms suggests a broader capacity to CO_2_ uptake and routing of fixed carbon to value-added products. A few innovative studies have come up recently for improving photosynthetic efficiency in diatoms. One of these strategies is the use of a high silicate medium along with blue light under high light conditions (255 µE m^−2^ s^−1^) for fucoxanthin production in *P. tricornutum* [145]. Another is the application of intracellular spectral recompositioning of light (ISR) on a genetically engineered *P. tricornutum*, with a green fluorescent protein (GFP) to enhance photosynthetic efficiency (by 50%) and biomass productivity, influencing fucoxanthin production in diatoms [146]. In addition, enhanced lipid production in genetically modified *P. tricornutum* stable strains has been achieved by the use of gene editing tools such as meganucleases and transcription activator-like effector nucleases (TALEN) to achieve targeted disruption of the UDP-glucose pyrophosphorylase gene, a step involved in carbohydrate accumulation that enabled routing carbon to lipid accumulation [147].

## 4. Opportunities and Challenges of CO_2_ Sequestration by Diatoms—Direct Air Capture, Pure CO_2_ or Flue Gases

CO_2_ is an indispensable resource for autotrophic organisms such as diatoms. Effective utilization of diatoms for CO_2_ sequestration in biomanufacturing requires in-depth consideration of issues, such as CO_2_ supply, CO_2_ source and the optimization of conditions for species-specific CO_2_ uptake. 

CO_2_ supply is an important aspect to be considered for growing autotrophic microorganisms. CO_2_ can be supplied in either the solid form (as carbonate or bicarbonate salts) or in the gaseous form to autotrophic organisms. There are three potential gaseous sources; (a) flue gases or product streams from industry, (b) purified CO_2_ available in cylinders and (c) direct air capture. Flue gas, a by-product of industrial production and power generation, can be a useful CO_2_ resource. Flue gas from cement manufacturing has been tested on the diatom *S. marinoi*, and found to be non-toxic. In fact, high quality of microalgal biomass (lipids 20–30% DW, proteins 20–28% DW, carbohydrates 15–30%(DW)) and a higher biomass productivity has been demonstrated with flue gas addition, compared to aeration with atmospheric level of CO_2_ [148]. Flue gas from industry (which might include SO_x_, NO_x_ and other gases along with CO_2_), typically contains CO_2_ in the range of 6–15%, whilst product streams, such as from ethanol manufacturing and biogas, typically contain CO_2_ in the range of 20–40%. CO_2_ can also be supplied to the diatoms directly from air (which contains ~0.04% CO_2_), in the form of pure CO_2_, or CO_2_ mixed with air or nitrogen in cylinders (available up to 100% CO_2_). A challenge in providing CO_2_ from air is in arriving at economically viable propositions for extracting the CO_2_ from air. A commercial plant, where CO_2_ is sucked from the air (before being resold), had opened up in Switzerland. It was founded by Climeworks (https://www.climeworks.com/); the direct air capture (DAC) plant is capable of removing 900 tons of CO_2_ from ambient air annually. Unlike capturing emissions from industrial flue stacks, the technology by Carbon Engineering (https://carbonengineering.com/) captures CO_2_ directly out of the air. From a pilot facility in Squamish, Canada they had fully demonstrated the Direct Air Capture (DAC) technology, and are now commercializing. The SOLETAIR project (https://soletair.fi/) is also involved in direct CO_2_ capture from the air. For a successful diatoms-based bio-venture, the project should be environment friendly, sustainable, feasible on a large scale, and preferably work around emerging technologies such as DAC. 

Diatoms are mostly cultivated either in submerged or immobilized reactors. There are many challenges in supplying CO_2_ for cultivation and administered by the bubbling method. CO_2_ bubbled into the medium needs to be dissolved and accessible to the diatoms. At high supply rates, and in saturated media, most of the CO_2_ supplied is released back into the atmosphere [84], due to the low CO_2_ solubility and low retention potential of CO_2_ in the medium. Moreover, the larger the bubble size, the greater the buoyancy and faster the release of CO_2_ bubbled out from the medium (and hence, lost from the system). The CO_2_ utilization of photoautotrophic organisms is also slow and limited. Factors which affect CO_2_ utilization efficiency and growth include CO_2_ concentration, bubble size, aeration rate, mixing time, and the residence time of the bubble. Ensuring an extensive air/liquid interface is essential for ensuring good CO_2_ mass transfer [149]. The CO_2_/O_2_ balance is also a key factor in attaining a higher photosynthetic rate. The knowledge of CO_2_ uptake in most of the diatom species is fragmentary. For CO_2_ fixation, CO_2_ can be injected as a gas into the culture, dissolved in a separate absorption column, or added as an alkaline solution in the form of bicarbonate [83]. Direct injection of CO_2_ is known to lower the pH of the culture, and can adversely affect growth and biomass productivity at high CO_2_ concentrations. There is a body of literature on high CO_2_ tolerance for cultivation of diatoms with improved product yields. For example, the addition of 10% CO_2_ (v/v) into the cultures of *T. weisflogii* and *C. cryptica* resulted in doubling the lipid content in comparison to air sparging, but induced only a modest increase of biomass. In the same set up, CO_2_ also stimulated lipogenesis in both of the diatoms (*T. weisflogii* and *C. cryptica*). Moreover, TAG became the major lipid component, and accounted for more than 60% of total glycerolipids in *C. cryptica*. [150]

Approaches such as microbubble generation [151] have enabled increased surface area for effective mass transfer in dissolving gaseous CO_2_ into the culturing medium. There are also a variety of cultivation systems that focus on the method of CO_2_ supply into the cultivation medium. The optimization of carbon use in pilot-scale outdoor tubular photo-bioreactors by application of effective control techniques, such as model-based predictive control (MPC), for reducing loss of CO_2_ along with total supply of CO_2_ volume, has been reported to potentially increase productivity by 15% and reduce the cost of producing biomass by >6% [152]. Different requirements may need to be considered for the supply of CO_2_ into open cultivation systems, such as lakes, lagoons, ponds, constructed raceway ponds and closed systems, such as tubular photo-bioreactors, flat panel photo-bioreactor, fermenters, cascade raceways, raceways and Tic bag photo-bioreactors. 

In its solid form, HCO_3_^−^ can be a source of carbon. Bicarbonate is the main form of inorganic carbon utilized by *P. tricornutum* [153]. Sodium bicarbonate is readily available in large quantities due to coal fired power stations, using a CO_2_ scrubbing system and generating bicarbonates. Bicarbonate is also easier to transport than gaseous CO_2_ [123]. Sodium has been found to increase the affinity for inorganic carbon and facilitate the utilization of bicarbonate in *P. tricornutum* [154]. Sodium ions can enhance the rate of photosynthetic oxygen evolution, which could be due to the presence of a sodium dependent bicarbonate-transport system, and as the internal inorganic-carbon concentration is lower in relation to the external concentration, the effect of sodium is possibly at the plasmalemma [95]. *P. tricornutum* has been shown to have a similar growth rate and CO_2_ uptake rate at extreme pH values compared with neutral pH [83]. Bicarbonate feeding was found to reduce the loss of CO_2_ to the environment compared with gaseous CO_2_ supply, however, the growth rate was reduced, along with the biomass yield [83]. Three different concentrations of NaHCO_3_ (5, 15 and 25 mM) have been added at one of two different culturing phases, either at day 0 (during bioreactor inoculation) or at day 4 (~24 h before nitrate depletion). The cultures supplemented with 15 mM NaHCO_3_ accumulated more carbohydrate than the control culture. The supplementation of 25 mM NaHCO_3_ led to higher protein content for unknown reasons (Mus et al. 2013). *Nitzschia plea* is known to tolerate high concentration of NaHCO_3_ (0.15 mol/L) and high pH (>10) [155].

A diatom that has the machinery for both HCO_3_^−^ uptake and CO_2_ capture will be indispensable for industrial applications, and enable the development of a sustainable biomanufacturing process. There are a few diatoms that are known to have the capacity to utilize both CO_2_ and HCO_3_^−^. *N. palea kutzing* is a very interesting diatom in this regard, and its cells were found to be capable of using HCO_3_^−^ in addition to gaseous CO_2_, and the CO_2_ enrichment decreased their affinity of HCO_3_^−^ and CO_2_ [156]. Another diatom, *Chaetocerous muelleri*, has the capacity to use bicarbonate to acquire inorganic carbon through one or multiple CCM. It also has the capacity to use HCO_3_^−^ to acquire inorganic carbon, through one or multiple pathways [157]. Storage of DIC is also an area that requires detailed study in diatoms, an example being the preference of HCO_3_^−^ by *Cyclotella* sp. and *Nitzschia* sp. [158]. 

The carbon capture potential of a diatom is directly related to its CCM design and the efficiency of its CCM enzymes, such as CAs and RuBisCO. The regulation of external CA activity and photosynthetic CO_2_ affinity are dependent not only on CO_2_ concentration, but also on light availability, as observed in *S. costatum*. The presence and activity of external CAs decide how well a CCM is designed. External CA activity has been detected in cells grown at 4 μmol L^−1^ CO_2_, but not at 31 and 12 μmol L^−1^ CO_2_, with its activity being about 2.5 times higher at high irradiance than at low irradiance. Further, the development of higher external CA activity and CO_2_ affinity under higher light level could sufficiently support the photosynthetic demand for CO_2_, even at a low level of CO_2_ [159]. Light has a pivotal role to play in CCM efficiency. At saturating light intensities, *S. costatum cleve* and *P. tricornutum Bohlin* maintain maximum photosynthetic rates under low CO_2_ levels, but *P. tricornutum* is well adapted to rapid changes in irradiance and CO_2_ availability. In *P. triconutum* and *Nitzschia ovalis*, acetate has been found to be the preferred carbon source for the formation of the sterols in the cytoplasm, via the mevalonate pathway. Also, CO_2_ was regarded as the main source for phytol biosynthesis in the chloroplasts, via the mevalonate independent methyl erythritol 4-phosphate pathway. Both the diatoms, *P. triconutum* and *N. ovalis*, have been found to display the same compartmentation for isoprenoid biosynthesis, as previously found in higher plants, the red alga *Porphyridium cruentum* and the chrysophyte *Ochromonas danica* [160]. The extracellular carbohydrates of the diatom *Cyclotella meneghiniana* have been found to increase with elevated CO_2_ and temperature [161]. Inlet pCO_2_ have been found to enhance lipid production along with chitin formation in *Cyclotella* sp. in a photobioreactor setup [162].

Along with the biology of a diatom, the carbon capture potential is also dependent on the culture health and viability. Moreover, the efficiency of its CCMs depends on the physical, chemical and biological conditions in the culturing environment. The amount of carbon captured in the system is directly proportional to the density and growth phase of the diatom. For diatoms, the literature on CO_2_ fixation is sparse. Buono et al. (2016) had found that there was no linear relationship between the CO_2_ added to the culture and the CO_2_ assimilated by the microalga [30]. A significant amount of CO_2_ was found to be lost to the atmosphere when the gas was added to the culture. It has been found that closed systems, compared with open ponds, have a better ability to assimilate CO_2_ (a 44.2% reduction in CO_2_ fixation was observed in open ponds) [30,149], and had resulted in higher CO_2_ fixation, biomass productivity, and a higher photosynthetic efficiency [30]. A high CO_2_ fixation rate in *P. tricornutum* was observed in 1 L cultures when supplemented with 15% (v/v) CO_2_, resulting in a CO_2_ fixation rate of 0.282 g L^−1^ d^−1^, but the biomass productivity was low (0.15 g L^−1^ d^−1^) [85]. pH plays an interesting role, as evident when the culture pH under 15% CO_2_ (pH 6.3) compared to a buffered system (pH 7) did not significantly affect the biomass productivity [85]. *P. tricornutum* takes up HCO_3_^−^ predominantly, whilst *T. pseudonana* takes up CO_2_ [86]. 

The species and strain of diatom to be used for large scale carbon capture needs to be able to grow under a day-night cycle, be suitable for large scale cultivation, and can be coupled directly with the CO_2_ flue gas from a power plant [149]. Utilizing flue gases for microalgal cultivation would be beneficial as, in addition to high CO_2_, it also contains NO_x_ and sulfur dioxide (SO_2_), that can be used by the diatom as a nitrogen and sulphur source. However, there is a problem associated with it, as shown for *P. tricornutum*, where the addition of SO_2_ at 50 ppm resulted in the growth being slightly inhibited, and a further increase to 400 ppm caused a cessation of growth [85]. When CO_2_ from flue gas is used, a high pH is required to ensure the bicarbonate remains in solution and does not dissipate into the atmosphere [83].

An ideal cultivation system would enable appropriate carbon supply, carbon uptake and CO_2_ fixation using diatoms. The construction of a suitable PBR appears to be essential for successful CO_2_ supply, reduction in CO_2_ wastage and carbon sequestration. Carbon sequestration by microalgae is itself dependent on characteristics of light (intensity, incident angle, photo period and wavelength) and carbon optimization, both required to improve photosynthesis for CO_2_ uptake. Open ponds are often employed worldwide, due to their economic benefit, but are constrained by low biomass productivities, issues with evaporation, CO_2_ diffusion to the atmosphere, a lack of temperature control, insufficient light transmission, and vulnerability to contamination. Comparatively, flat-plate and tubular PBRs have been shown to have a large surface area, good mixing, mass transfer and short internal light paths. However, tubular PBR systems can be constrained in their size and length, because of oxygen build up and CO_2_ depletion [149]. Air-lift PBRs have been reported to have a higher CO_2_ fixation rate, due to their better circulation and mass transfer through the use of risers and downcomers [163], but bubbles can cause a high attenuation of light and can create shear when they break. Several strategies have been employed, like degassers, air outlets through water traps etc. in PBRs to strip this excess oxygen from the culture medium with air or inert gases [164]. However, more efficient methods are required to alleviate the effects of O_2_ inhibitory effects during microalgal growth. *P. tricornutum* has been successfully cultivated on a large scale (55 L) in a flat-plate customized photobioreactor, for the simultaneous synthesis of storage lipids, EPA, fucoxanthin and chrysolaminarin [56]. *P. tricornutum* has also been cultivated outdoors in 800 L bubble column PBR [165], and in 1250 L indoor open raceway pond [58].

## 5. Bio-Manufacturing with CO_2_ Uptake

One of the diatoms which has been extensively evaluated for potential as a bio-based manufacturing chassis is *P. tricornutum*, a versatile diatom that has the capacity to produce a range of natural (fucoxanthin, EPA, DHA, oil, brassicosterol, and chrysolaminarin) and genetically engineered products (lupeol, betulin, arachidonic acid, antibodies, and polyhydroxybutyrate) [23]. It is a well characterized diatom, and can be routinely cultivated in the laboratory, and has been shown to perform well at scale (>1250 L) [58]. *P. tricornutum* is a saltwater diatom and offers potential as a sustainable cell chassis for multiple products of interest, and capable of performing well in constrained environments, including low light and high pH [83,166]. It has a relatively well annotated genome and demonstrated cases of downstream processing for the sequential extraction of multiple products of interest using a bio-refinery approach [155].

Another diatom, *Chaetocerous gracilis*, has been shown to accumulate TAG without nutrient deprivation, and has a great potential as a biofactory [167]. CO_2_ addition is associated with increased protein content and lowered carbohydrates, but had no effect on lipid content in the marine diatom *Chaetoceros* cf. *wighamii* [168]. 

The marine diatoms, as a group, have potential for high CO_2_ fixing capacity, being naturally evolved for this function. However, further elucidation of carbon fixation is required before strategies can be developed to maximize carbon uptake and route fixed carbon to products of value in industrial scale operations. Our knowledge of molecular pathways and strategies to improve carbon uptake are rudimentary at this stage, and will need to be developed for more diatoms than has been available so far. More information will enable ways to maximize CO_2_ uptake and route it effectively, to increase the productivity of diverse products. Combined mixotrophic approaches might help further. Improvements in cultivation, PBR development, and strategies, including adaptive laboratory evolution, omics analyses and targeted genetic engineering, will be useful in taking the investigations forward towards sustainable implementations. 

The steady states of specific intracellular levels of carbon metabolic intermediates affect the yield of bio-based manufacturing products, and can be increased by higher amounts of CO_2_ concentrations. Acetyl CoA is the precursor for lipids, carotenoids, exopolysaccharides (EPS) and other valuable metabolites in most organisms, and shunting carbon through this metabolite would be a useful way to elevate product yields. Supplementation of 2.6% CO_2_ has been shown to lead to increased Acetyl CoA in *P. tricornutum*, with a 41, 25% and 27% increase over the air-sparged controls in the lag, log and stationary phases, respectively [143]. Acetyl CoA (AcCoA) has also been shown to increase with exposure to increased CO_2_ concentrations, and in turn lead to improved lipid accumulation [169]. However, long term exposure to elevated pCO_2_ has been shown to have a detrimental effect on the diatom *Cylindrotheca fusiformis* [170]. Carbohydrate yields in different EPS fractions increased with elevated pCO_2_ exposure. Although the proportions of monosaccharide sugars among total sugars did not change, higher abundances of uronic acid were observed under high pCO_2_ conditions, suggesting the alteration of EPS composition [171]. An increase in CO_2_ supplied from 400 ppm to 20,000 ppm has been shown to lead to a general increase in biomass productivity, by 11%, 28% and 21%, respectively, in *T. pseudonana*, *P. tricornutum* and *N. pelliculosa* (seawater strain), in the exponential phase, when other nutrients are expected to still be available in sufficient quantities [28]. Such positive results open up new avenues for CO_2_ capture.

There are many advantages of culturing diatoms for large scale cultivation, especially those from the marine habitat. Sea water can be used as the culturing medium, thereby preventing the usage of scarce freshwater resources. Diatoms are robust organisms, competitive, and can be cultivated in less stringent and non-sterile conditions. They are encased with a silica layer that helps them to evade predators and grazers [172], except the less silicified oval stage of *P. tricornutum* [173,174]. The strong silica frustule may also help them to maintain structural integrity in the rough seas, and provide buoyancy for the cells to access nutrient and light enriched surfaces [175]. This can also create density, enabling implementable harvesting protocols. However, exposure to pests leading to cultivation crashes and subsequent economic losses still require addressing and the development of innovative solutions. With an ever-increasing human population, more food will need to be produced and more energy will be consumed as a result. In a transition to a bio-based economy with sustainable solutions, diatoms have the potential to be the futuristic and sustainable source for CO_2_ sequestration and bio-based manufacturing.

## 6. Conclusions

Diatoms are responsible for 20% of the global CO_2_ fixation, a major greenhouse gas responsible for climate change. In addition to sequestering CO_2_, diatoms can be utilized to produce a plethora of commercially viable products for food, feed, fuel, and nutraceuticals/pharmaceuticals applications. There is a diversity of products with market potential, ranging from high value low volume to low value high volume productions. In order to accommodate the diverse requirements of value addition and still be effective contributors to CO_2_ mitigation, the development of strategies along the biorefinery concept will be necessary. Much of our knowledge on diatoms for use in carbon sequestration and biomanufacturing has been built on a handful of species, *P. tricornutum* being the dominant one. Carbon metabolism in more diatom species requires elucidation, especially with respect to exposure to industrial CO_2_ supply compared to the natural environment, and the bottlenecks in the CCM and the overall carbon capture pathways understood. These remain as challenges to be overcome for diatoms to be developed as a microbial cell factory for a suite of products of commercial interest, whilst simultaneously making the most of their CO_2_ utilizing capacity for CO_2_ sequestration strategies. There is a huge potential in diatoms for carbon capture and utilization and in bio-based manufacturing that awaits research and development.

## Figures and Tables

**Table 1:** Exemplar products from diatoms for bio-manufacturing, using autotrophy or mixotrophy.

Products for Biomanufacturing	Species	Product Yield/Productivity Reported	Reference
Antibacterial substances	*Phaeodactylum tricornutum* EPSAG	*n/a*	[45]
Arachidonic acid	*Phaeodactylum tricornutum* CCAP 1055/1 (recombinant)	1.89% DW; 22% TFAs	[46]
	*Cylindrotheca fusiformis* UTEX 2084	8.19% TFAs	[47]
	*Nitzschia* sp. FD397	0.3% DW; 2.24% TFAs	[48]
	*Nitzchia palea*	21.61% TFAs	[49]
Biomass	*Phaeodactylum tricornutum* UTEX 640	25.4 g/L; 1.7 g/L/d	[50]
	*Amphora* sp. MUR258	0.171 g/L/d	[51]
	*Chaetoceros* sp.	0.125 g/L/d	[52]
	*Skeletonema* sp.	0.185 g/L/d	[52]
	*Thalassiosira* sp.	0.312 g/L/d	[52]
	*Thallasiosira weissflogii*	3.83 g/m2/d	[53]
	*Skeletonema* sp. UHO29	0.34 g/L/d	[54]
	*Nitzchia laevis* UTEX 2047	0.4 g/L/d	[55]
Chrysolaminarin	*Phaeodactylum tricornutum* CAS	94 mg/L/d; 14% DW	[56]
	*Odontella aurita* SCCAP K 1251	161.55 mg/L/d	[57]
Docosahexaenoic acid (C22:6, *n*-3)	*Phaeodactylum tricornutum* CCAP 1055/1 Pt_El05 (recombinant)	0.64% DW	[58]
Eicosapentaenoic acid (C20:5, *n*-3)	*Nitzchia laevis* UTEX 2047	10.46 mg/L/d; 19.15% DW	[55]
	*Fistulifera solaris* JPCC DA0580	135.7 mg/L/d; 11.7% DW; 38.6% TFAs	[59]
	*Thalassiosira weissflogii*	33.4 mg/L/d; 24.2% TFAs	[60]
	*Odontella aurita* SCCAP K 1251	9.37 mg/L/d; 25.3% TFAs	[57]
	*Cyclotella cryptica* CCAP 1070/2	3.8 % DW; 14.4% TFAs	[61]
	*Cylindrotheca fusiformis* UTEX 2084	24.63% TFAs	[47]
	*Phaeodactylum tricornutum* UTEX 640	56 mg/L/d; 3.29% DW	[50]
Extra polymeric substances	*Phaeodactylum tricornutum*	*n/a*	[62]
Fucoxanthin	*Nitzchia* sp. KMMCC-308	0.492% DW	[63]
	*Mallomonas* SBV13	2.66% DW	[64]
	*Phaeodactylum tricornutum* CAS	4.7 mg/L/d; 0.7% DW	[56]
	*Phaeodactylum tricornutum* CS-29	2.28 mg/L/d; 5.92% DW	[65]
	*Odontella aurita SCCAP* K 1251	6.01 mg/L/d; 2.33% DW	[57]
	*Chaetoceros gracilis* KMMCC-27	0.223% DW	[63]
	*Thalassiosira weissflogii*	0.95% DW	[60]
	*Odontella aurita* SCCAP K-1251	2.17% DW	[66]
	*Cylindrotheca closterium*	0.523% DW	[67]
Triacylglycerols (TAGs)	*Cylindrotheca fusiformis* CCAP 1017/2	7.2 mg/L/d; 24.5% DW	[61]
	*Chaeotoceros muelleri* CCAP 1010/3	5.2 mg/L/d; 23.9% DW	[61]
	*Chaetoceros simplex* CCAP 1085/3	5.2 mg/L/d; 19.6% DW	[61]
	*Amphora* sp. MUR258	62 mg/L/d (lipid); 36.26% DW	[51]
	*Phaeodactylum tricornutum*	58.5 mg/L/d; 45% DW	[68]
	*Thalassiosira weissflogii* P09	3.7 mg/L/d; 15% DW	[69]
	*Thalassiosira weissflogii* CCMP 1010	2.58 mg/L/d; 21% DW	[69]
	*Thalassiosira weissflogii* CCMP 1336	1.57 mg/L/d; 11% DW	[69]
	*Thallasiosira psuedonana* CCMP 1335	0.33 mg/L/d; 6% DW	[69]
	*Navicula pelliculosa*	21.4% DW	[70]
	*Nitzschia closterium*	38.8% DW	[70]
	*Nitzschia longissima*	25.8% DW	[70]
	*Nitzschia ovalis*	21% DW	[70]
	*Nitzschia frustulum*	11.8% DW	[70]
	*Amphora exigua*	23.6% DW	[70]
	*Amphora* sp.	18.1% DW	[70]
	*Biddulphia aurica*	19.3% DW	[70]
	*Fragilaria* sp.	11% DW	[70]
	*Chaetoceros* sp.	10.2% DW	[71]
	*Cyclotella cryptica* CCAP 1070/2	4 mg/L/d; 23.5% DW	[61]
	*Cyclotella cryptica* CCMP 331	1.64 mg/L/d; 23.06% DW	[69]
Oxylipins	*Cocconeis scutellum* parva	*n/a*	[72]
	*Skeletonema marinoi*	*n/a*	[72]
	*Skeletonema costacum*	*n/a*	[72]
	*Chaetoceros pseudocurvisetus*	*n/a*	[72]
Phytosterol/Sterol	*Chaetoceros muelleri*	0.4% DW (fucosterol), 0.25% DW (cholesterol)	[73]
	*Phaeodactylum tricornutum*	0.5% DW (brassicasterol)	[73]
	*Thalassiosira pseudonana*	0.25% DW (24-Methylenecholesta-5, 24(24′)-dien-3Beta-ol	[73]
Silica		*n/a*	[74]
Sulfated polysaccharides	*Phaeodactylum tricornutum*	20.15 mg/L/d	[75]
Polyhydroxybutyrate (PHB)	*Phaeodactylum tricornutum* CCAP 1055/1	10.6% DW	[76]
Human igGαHBSAg	*Phaeodactylum tricornutum* UTEX 646	0.0021% DW (8.7% total soluble protein)	[77]
IgG1/kappa ab CL4mAb	*Phaeodactylum tricornutum* UTEX 646	2.5 mg/L (secreted)	[78]
Monoclonal IgG antibodies against the nucleoprotein of Marburg virus	*Phaeodactylum tricornutum* UTEX 646	2 mg/L (secreted)	[79]

## Abbreviations

CO_2_	carbon dioxide
CCM	carbon concentrating mechanism
CA	carbonic anhydrase
RuBisCO	ribulose-1,5-bisphosphate carboxylase/oxygenase
PBR	photobioreactor
TAG	triacylglycerol
DIC	dissolved inorganic carbon
SLC4	solute carrier 4
AQPs	aquaporins
